# Modulation of functionally localized right insular cortex activity using real-time fMRI-based neurofeedback

**DOI:** 10.3389/fnhum.2013.00638

**Published:** 2013-10-10

**Authors:** Brian D. Berman, Silvina G. Horovitz, Mark Hallett

**Affiliations:** ^1^Department of Neurology, University of Colorado Anschutz Medical CampusAurora, CO, USA; ^2^Human Motor Control Section, National Institute of Neurological Disorders and StrokeBethesda, MD, USA

**Keywords:** neural modulation, real-time fMRI, biofeedback, insular cortex, urges

## Abstract

The capacity for subjects to learn to volitionally control localized brain activity using neurofeedback is actively being investigated. We aimed to investigate the ability of healthy volunteers to quickly learn to use visual feedback during real-time functional MRI (rtfMRI) to modulate brain activity within their anterior right insular cortex (RIC) localized during a blink suppression task, an approach of possible interest in the use of rtfMRI to reduce urges. The RIC region of interest (RIC-ROI) was functionally localized using a blink suppression task, and blood-oxygen level dependent (BOLD) signal changes within RIC-ROI used to create a constantly updating display fed back to the subject in the scanner. Subjects were instructed to use emotional imagery to try and increase activity within RIC-ROI during four feedback training runs (FB1–FB4). A “control” run (CNTRL) before training and a “transfer” run (XSFR) after training were performed without feedback to assess for baseline abilities and learning effects. Fourteen participants completed all neurofeedback training runs. At the group-level, increased BOLD activity was seen in the anterior RIC during all the FB runs, but a significant increase in the functionally defined RIC-ROI was only attained during FB2. In atlas-defined insular cortex ROIs, significant increases were seen bilaterally during the CNTRL, FB1, FB2, and FB4 runs. Increased activity within the insular cortices did not show lateralization. Training did, however, result in a significant increase in functional connectivity between the RIC-ROI and the medial frontal gyrus when comparing FB4 to FB1. Since neurofeedback training did not lead to an increase in BOLD signal across all feedback runs, we suggest that learning to control one’s brain activity in this fashion may require longer or repeated rtfMRI training sessions.

## INTRODUCTION

Technological advances in computer hardware and functional MRI (fMRI) data processing software have made it possible to analyze neural activity as measured by changes in blood-oxygen level dependent (BOLD) contrast almost as quickly as images are acquired. This real-time fMRI (rtfMRI) approach allows for the displaying of measures of localized brain activity back to a subject in a scanner and investigation of their ability to learn to volitionally control their own brain activity (deCharms, 2007; Weiskopf et al., 2007; Weiskopf, 2012). The use of such rtfMRI-guided neurofeedback offers significant advances over traditional biofeedback with evaluation involving whole brain coverage, good spatial resolution, and ability to target specific brain regions in a given patient.

An increasing number of rtfMRI studies have been reported suggesting that healthy subjects can learn through operant training to use neurofeedback to control the activity in a wide range of cerebral regions. These regions include the anterior cingulate cortex (Weiskopf et al., 2003; deCharms et al., 2005), right inferior frontal gyrus (Rota et al., 2008), and auditory cortex (Yoo et al., 2006), as well as the difference between activation in supplementary motor area and parahippocampal place area (Weiskopf et al., 2004) and in motor-associated cortices during motor tasks (Posse et al., 2001; Yoo and Jolesz, 2002) and during motor imagery tasks (deCharms et al., 2004; Yoo et al., 2004, 2007, 2008; Berman et al., 2012b). In addition to these brain regions, rtfMRI-based modulation of limbic-associated brain regions has also been demonstrated in neurofeedback studies involving the amygdala (Posse et al., 2003; Zotev et al., 2011) and insular cortex (Caria et al., 2007, 2010; Johnston et al., 2010; Ruiz et al., 2011; Veit et al., 2012).

Recently, rtfMRI-based neurofeedback has demonstrated the potential to lead to clinical effects in certain patient populations. Preliminary studies suggest neurofeedback may have benefit in patients suffering from chronic pain (deCharms et al., 2005), tinnitus (Haller et al., 2010), depression (Linden et al., 2012), and Parkinson disease (Subramanian et al., 2011). One study found that patients with schizophrenia showed improved performance on a face recognition task after neurofeedback training focused on modulating insula activity (Ruiz et al., 2011). Although Tourette syndrome has shown some success in treatment with biofeedback and EEG-guided neurofeedback training (Tansey, 1986; O’Connor et al., 1995; Piacentini and Chang, 2001; Heinrich et al., 2007; Messerotti Benvenuti et al., 2011), the use of rtfMRI-guided neurofeedback to treat Tourette syndrome has, to the best of our knowledge, not been reported.

A number of imaging studies from our lab and other investigators have supported the presence of abnormal limbic-motor coupling in patients with the neuropsychiatric disorder Tourette Syndrome (Jeffries et al., 2002) as well as involvement of the insular cortex during tic initiation and execution (Stern et al., 2000; Bohlhalter et al., 2006; Lerner et al., 2007). Given the association of insula activity with tic generation in Tourette syndrome, and since tic performance is frequently preceded by a premonitory urge (Kwak et al., 2003), learned modulation of insular cortex activity through rtfMRI-guided neurofeedback training could provide an effective approach by which patients could learn to consciously inhibit the onset of a tic. The insular cortex might also be an especially good target for self-modulation as it has been shown to be involved in a wide range of functions including sensory perception and integration, motor control, and emotive and cognitive functioning, in addition to self-awareness and interpersonal experience (Craig, 2002).

In the present study, we sought to develop and establish an rtfMRI-based neurofeedback training methodology that could be used for future investigation as a therapeutic intervention in neuropsychiatric conditions associated with disordered suppression where a role for the insular cortex has been implicated such as Tourette syndrome (Bohlhalter et al., 2006; Lerner et al., 2007; Fahim et al., 2009), obsessive–compulsive disorder (Nishida et al., 2011; Stern et al., 2011), eating disorders (Kim et al., 2012; Lawson et al., 2012), and post-traumatic stress disorder (Nagai et al., 2007; Herringa et al., 2012). We first chose to specifically target the anatomic region of the anterior right insular cortex (RIC), which supports a representation of visceral responses thought to be accessible to awareness (Critchley et al., 2004). We employed an eye blink suppression task to refine the location of the targeted region for neurofeedback to an area that is associated with urge suppression (Berman et al., 2012a). Blink suppression was used for functional localization because blinking is often one of the earliest manifestations and most common tics in Tourette syndrome and because the buildup of the urge to during blink inhibition and the relief that accompanies their eventual performance can serve as a model for the buildup of uncomfortable sensations that commonly precede tics (Shapiro et al., 1988; Peterson and Leckman, 1998).

Given that the preferential recruitment of the insula during tasks involving recall and imagery of emotionally relevant events (Phan et al., 2002), along with the success of recent rtfMRI studies involving modulation of right anterior insular cortex activity with thoughts with emotional valence (Caria et al., 2007, 2010), our study participants were instructed to use cognitive strategies that focused on emotional induction by recall or imagery of emotionally relevant events during neurofeedback training. We hypothesized that healthy volunteers would be able to learn how to self-modulate neural activity within their anterior RIC that is functionally localized to a region specifically involved during the suppression of blinking using rtfMRI-guided neurofeedback.

## MATERIALS AND METHODS

### PARTICIPANTS

We enrolled a total of sixteen healthy volunteers, aged 29.3 ± 7.8 years (9F, 7M). All participants had normal neurological examinations and all but one were right-handed by the Edinburgh Handedness Inventory (Oldfield, 1971). The study was approved by the Combined Neurosciences Institutional Review Board of the National Institutes of Health, and all participants gave their written informed consent before participation.

### IMAGING DATA ACQUISITION

Images were acquired with a 3T scanner and 8-channel head coil (GE Signa, Milwaukee, WI, USA) foam-padded to restrict head motion and improve subject comfort. Functional T2*-weighted images were acquired using gradient echo, echo planar imaging using the imaging acquisition parameters: matrix size = 64 × 64, field of view (FOV) = 22 cm × 22 cm, TR = 1000 ms (800 ms for first six subjects), TE = 30 ms, flip angle = 70°, bandwidth = 250 kHz. Each scan consisted of 14 or 17 slices that covered most of the brain except for the cerebellum (3.3 mm × 3.3 mm nominal in-plane resolution, 5.0 mm thick slices, 0.5 mm gap). High-order shimming was applied to lessen the field inhomogeneities during data collection and improve the signal-to-noise ratio in areas prone to susceptibility artifacts. A high-resolution magnetization-prepared rapid gradient echo anatomical scan was acquired for each subject for superposition of functional maps upon brain anatomy and to allow for image normalization to a standardized brain space (matrix size = 256 × 256, FOV = 22 cm × 22 cm, 1 mm^3^ isotropic resolution, TR = 10 ms, TE = 4.96 ms, flip angle = 19°).

### FUNCTIONAL LOCALIZATION OF ANTERIOR RIGHT INSULAR CORTEX

**FIGURE 1:**
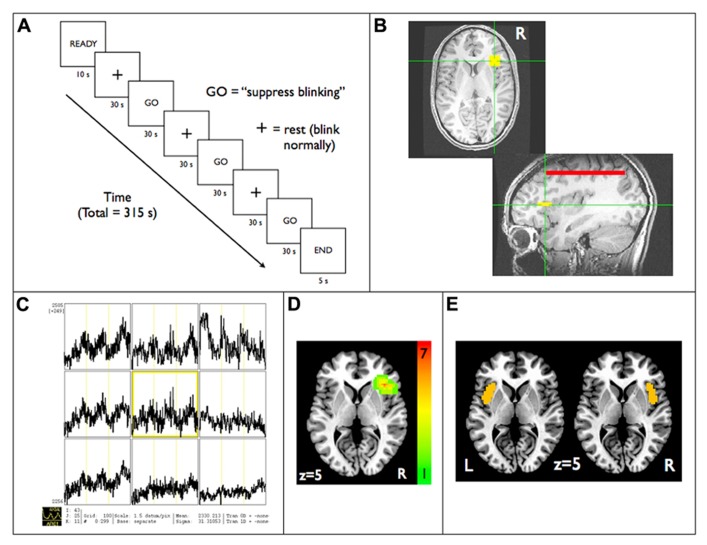
****Functional localization of the anterior RIC: **(A)** real-time fMRI scanning paradigm used for blink suppression task. RIC-ROI (5 × 5 voxels, yellow square) is first **(B)** localized by using anatomical landmarks and then **(C)** centered on the axial slice and voxel that maximized increases in BOLD signal within the ROI that corresponded temporally to the blink suppression blocks. Also shown is **(D)** a composite map that demonstrates the functionally localized ROIs were clustered around anatomical location of RIC and **(E)** the left and right anterior short insular gyrus anatomical ROIs defined using the Destrieux atlas, both displayed on standard axial brain slices in Talairach space. Red line shown in **(B)** is a reference ROI encompassing the entire brain volume in an axial slice distant to the RIC-ROI used in generating the neurofeedback display (see text). RIC, right insular cortex; ROI, region of interest.

Real-time fMRI data were acquired and exported in real-time to a console at the scanner running Analysis of Functional NeuroImages (AFNI) software (Cox, 1996), which allowed for real-time motion correction and monitoring of a continuously updating BOLD signal time-course display. During the functional localization scanning run, participants were instructed to inhibit eye blinking during three 60-s time periods (**Figure 1A**). Simultaneous electrooculography was used to ensure subjects were suppressing blinking (see methods in Berman et al., 2012a). A 5 × 5 voxel (16.5 mm × 16.5 mm) square region of interest (ROI) in the axial plane was initially positioned through the use of anatomical landmarks such that it was placed in the area of the anterior RIC (**Figure 1B**). BOLD signal responses during the blink suppression run were then explored in all three dimensions in the vicinity of the anatomically derived position of the ROI until a position was found that visually led to the maximum amount of buildup in BOLD signal in the ROI during the blink suppression blocks (**Figure 1C**). A composite map of the RIC-ROI for all subjects, created by summing RIC-ROI masks with each assigned a value of 1 and co-registered to a standard stereotactic space (Talairach and Tournoux, 1988), revealed a distribution of the ROIs clustered around the anatomical location of the RIC with maximum overlap of seven subjects’ RIC-ROI masks (**Figure 1D**).

### REAL-TIME NEUROFEEDBACK DISPLAY

A reference ROI (REF-ROI) encompassing the entire brain volume in an axial slice distant to the insular cortex ROI (see example red line, **Figure 1B**) was used to average out any unspecific activation and cancel out non-specific activation and global scanning effects. The mean BOLD signal within the specified ROIs were extracted and exported in real-time to a dedicated Linux workstation. In-house Python routines were developed to read BOLD signal changes, perform basic mathematical operations, and produce a dynamic visual display that conforms to standard block fMRI experimental design. The feedback display consisted of a red column with a height that was continuously updated after an initial baseline rest block at each TR using the following equation:

$$Column\,\;height\,\;\;(TR)=\frac{\left[RIC-ROI(TR)/RIC-ROI(baseline)\right]}{\left[REF-ROI(TR)/REF-ROI(baseline)\right]}$$

The feedback display also contained a solid bar at the top to represent the target level of activity, a dashed line representing the average level of activity measured during the baseline rest block, and an arrow to emphasize the direction brain activity is to be modulated.

### NEUROFEEDBACK TRAINING

**FIGURE 2:**
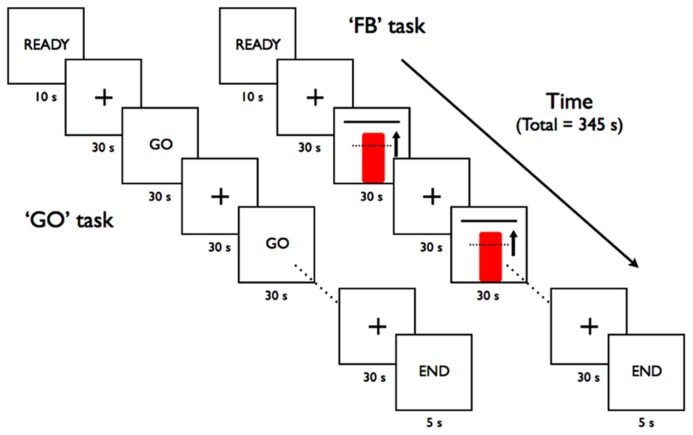
**Block-design task paradigms for the rtfMRI-based scanning runs**.

Two types of scanning runs were used during neurofeedback training with tasks presented in a block-design fashion (**Figure 2**). During the feedback runs (“FB”), participants were shown a continuously updated feedback display and instructed to increase the red column’s height toward the goal bar by focusing their thinking on recall or imagery of emotionally relevant events – a mental task based on a previously reported demonstration of subjects to use this strategy during neurofeedback training to modulate activity within the anterior RIC (Caria et al., 2007, 2010). The specific verbal instruction given to each subject to help guide their feedback strategies was for the subject to “focus on imagery or recall of emotionally relevant thoughts or memories.” During the “GO” runs, participants only saw the word GO on the screen and were instructed to perform the emotional imagery task in the absence of any visual feedback.

Each scanning run began with 10 s for scanner signal stabilization and participant acclimation to the scanner environment before an initial 30 s baseline rest block. The active GO and FB blocks were alternated with rest blocks during which participants were encouraged to relax and think of the letters “A, B, C” or numbers “1, 2, 3” in order to divert their focus from the emotional mental imagery used during the active blocks of the scanning runs. Each scanning run consisted of a total of the initial baseline rest block followed by five active regulation blocks (GO or FB) separated by rest blocks of 30 s each (**Figure 2**). A final 30 s rest block allowed the delayed hemodynamic response from the final active block to be included in the analysis.

Immediately prior to scanning, participants were instructed in the layout of the scanning runs outlined above and informed of the inherent hemodynamic delay in addition to an approximate additional 1 s delay required to process imaging data and update the neurofeedback display. Scanning runs for each subject consisted of an initial pre-training “control” GO run (CNTRL), followed by four FB training scanning runs (FB1–FB4), and a final post-training or “transfer” GO run (XSFR). The CNTRL run was performed to evaluate the ability of participants to modulate activity within the RIC-ROI before neurofeedback training and the XSFR task was performed to evaluate if neurofeedback training led to the ability of participants to modulate brain activity in the RIC-ROI without the presence of neurofeedback. After each of the scanning runs, participants were asked to briefly describe the type of emotional imagery they used during the previous run (with or without feedback). If the emotional imagery was of a personal nature, participants were informed they did not have to answer the question.

### OFF-LINE IMAGE ANALYSIS

Images were analyzed *post hoc* using AFNI and the *afni_proc.py* processing stream. The first 10 scans of each session were excluded from data analysis to account for T1 equilibration effects and subject scanner acclimation. Functional scanning images were corrected for motion and realigned using the last scan as a reference (closest to anatomical scan acquisition). Images were spatially smoothed using an isotropic 8-mm FWHM Gaussian filter to accommodate individual anatomical variability. The realigned images were co-registered to the high-resolution anatomical images and subsequently transformed into Talairach space (Talairach and Tournoux, 1988).

Task-related changes in BOLD signal at the individual level were estimated at each voxel using a block-design function convolved with a standard gamma-aviate hemodynamic response function and a general linear model (GLM). Covariates derived from motion parameters were included into the GLM to take into account artifacts caused by head motion. Group-level analysis was performed using a simplified mixed-effects model (one-sample *t* test) to test for within-group differences in task-related changes in BOLD. Family-wise error (FWE) correction for multiple comparisons was performed using Monte Carlo-based simulations with the AFNI program *3dClustSim*. We set overall significance at *p* ≤ 0.01 FWE corrected by using a voxel threshold of *p* ≤ 0.005 and a cluster size threshold of 113 voxels.

### ROI ANALYSIS

The functionally localized RIC-ROI for each subject was used to perform hypothesis-driven ROI analysis for each neurofeedback scanning run. BOLD times series used for the ROI analysis were extracted from imaging data that had undergone the same preprocessing steps as used for the whole brain analysis. The mean percent signal change between the active and rest blocks for all GO and FB runs was calculated for each subject separately and then averaged across subjects. Group-level analysis also included an evaluation of training effects using a one-way repeated measures ANOVA (Prism 6.0) to assess treatment effect across all six training runs and across the four FB training runs across subjects, and paired *t* tests for XSFR vs. CNTRL and FB4 vs. FB1. Significant increases in mean percent BOLD signal above a resting baseline for each neurofeedback run were also evaluated using paired *t* tests. Significance level threshold for the *t* tests and repeated measures ANOVA was set at *p *≤ 0.05.

Exploratory ROI analyses were also performed including assessing the “best performers” and the “best performances.” The “best performers” were evaluated because it is unlikely that all participants are able to quickly learn to use rtfMRI-guided neurofeedback to modulate brain activity within a relatively short training period. The “best performances” were evaluated to assess if a particular themes in the types of emotional imagery found to be most successful in increasing the activity in RIC-ROI could be identified. The “best performers” group included those participants who had a significant increase in BOLD signal during neurofeedback blocks for at least two of the four FB runs. The “best performances” included the top third performances for each neurofeedback scanning run independent of the participant. Paired and one-sample *t* tests were used to evaluate XSFR vs. CNTRL, FB4 vs. FB1, and increases in mean percent BOLD signal above a resting baseline for each neurofeedback run for the “best performers” and “best performances,” respectively. The emotional valence of each subject’s self-reported emotional imagery was also used to test whether negative or positive emotional valence was associated with better neurofeedback performance. Details of self-reported imagery were further assessed for similar themes and grouped in order to compare the effects of specific mental strategies in their ability to lead to significant increases in RIC-ROI over baseline during the neurofeedback runs. Significance level for *t* test comparisons was set at a threshold of *p *≤ 0.05.

Structurally defined right and left anterior insular cortex ROIs were used to investigate BOLD signal changes within the greater anterior insular cortex volumes during neurofeedback and to assess for laterality effects during neurofeedback training. These ROIs were defined using the anterior short insular gyrus as derived from probabilistic labeling of the SPM () single subject average image based on the Destrieux atlas in Freesurfer (). Both ROIs were smoothed using an 8 mm FWHM kernel analogous to that used for the rtfMRI data analysis and then intensity filtered to limit the overall size of the ROI, approximate the structures in Talairach space, and minimize artifactual increases in statistical thresholds due to large surface areas relative to volumes (see **Figure 1E**). A lateralization index (LI) was calculated for each subject using a normalized difference between percent signal change extracted from the target (%RIC) and contralateral ROI (%LIC) using the equation: LI = (%RIC - %LIC)/(%RIC+%LIC), as has been applied elsewhere (Caria et al., 2007).

### CONNECTIVITY ANALYSIS

The residual BOLD times series from the whole brain analysis were used for the connectivity analysis to assess whether neurofeedback training altered underlying connectivity between RIC-ROI and another brain region. For each run and each subject, the time series of the functionally localized RIC-ROI was used as seed and correlated with each voxel in the brain. Individual correlation maps were then transformed into Talairach space (Talairach and Tournoux, 1988), and *r* values were Fisher transformed to z-scores before performing group analysis. Group-level connectivity maps for each of the neurofeedback training runs were generated. Voxel-wise connectivity changes were then investigated between CNTRL run and the XSFR run and between FB1 and FB4. Overall significance was set at *p* ≤ 0.01 FWE corrected by using a voxel threshold of *p* ≤ 0.005 and a cluster size threshold of 113 voxels. We then extracted cluster statistics for each subject and each run using a mask generated from the significant clusters identified in the group-level connectivity map and tested them for changes using a repeated measures one-way ANOVA and paired *t* tests (Prism 6.0).

## RESULTS

### PARTICIPANTS

One participant was unable to remain still during the neurofeedback runs and had to have her scanning terminated. Technical scanner issues forced neurofeedback scanning to be terminated shortly after starting neurofeedback training in another participant. Thus, 14 participants (aged 29.7 ± 8.2 years; 8F, 6M) completed all four FB runs were included in the final analysis. Software glitches resulted in two of the14 participants not having usable CNTRL runs and one not having a usable XSFR run such that 12 CNTRL runs and 13 XSFR runs were available for final analysis.

### FUNCTIONAL LOCALIZATION OF ANTERIOR RIC

Fifteen participants completed the functional localizer blink suppression scanning and simultaneous electrooculography confirmed all participants were attempting to suppress blinking during functional localization run (see Berman et al., 2012a). Regions were identified for all participants within the vicinity of the anterior RIC that exhibited signal increasing BOLD signal responses consistent with the blink suppression blocks (see **Figure 1C**).

### NEUROFEEDBACK TRAINING

**FIGURE 3:**
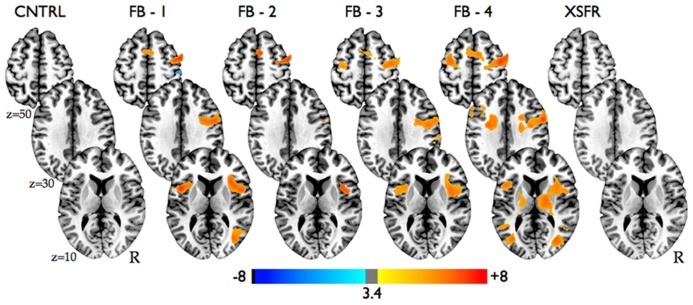
**Statistical parametric maps showing significant activation changes from baseline during all neurofeedback training runs in all participants (*n* = 14).** Images are shown at *p* ≤ 0.01, FWE corrected, on axial slices of a standard brain in Talairach space.

Group-level voxel-wise analysis (**Figure 3**, **Table 1**) revealed significantly increased BOLD activity within the region of the anterior RIC during all four training scanning runs when visual neurofeedback was provided (FB1–FB4), but not when there was no visual feedback (CNTRL and XSFR). Reported cognitive strategies employed by participants included both positive mental imagery (e.g., walking through the woods, lying on a beach, traveling, planning a party, and imaging actions of a character in a book), as well as negative imagery (recalling emotional or bad memories, remembering an argument, and focusing on someone close dying). Cognitive strategies that were associated with the best performances during neurofeedback training included both negative and positive mental imagery. Examples of the most successful thoughts were rather negative including thoughts of exerting an extreme effort, details of friend’s death and sadness, hunger and confinement, emotional memories, painful emotional experiences, pain in body parts; however, some of the most successful thoughts were positive including fond and hometown memories, sipping tea, and hearing a pleasant song.

**Table 1:** Brain areas with significant activation changes during neurofeedback training.

Task	Cluster size (voxels)	Side	Region (Brodmann area)	Talairach coordinates			Peak *t* value
				*X*	*Y*	*Z*
CNTRL	383	R	Precuneus (19), inferior parietal lobe (40)	32	-70	38	-6.06
FB 1	1049	R	Precentral gyrus (4,6), anterior insula, inferior frontal gyrus (6,44)	50	-4	44	7.74
	407	L/R	Medial frontal gyrus (6)	-10	-1	62	6.76
	308	L	Anterior insula	-40	8	5	7.00
	220	R	Middle occipital gyrus (19)	35	-64	5	6.45
	168	R	Postcentral gyrus (3)	38	-31	56	-4.45
FB 2	459	R	Precentral gyrus (6), anterior insula, inferior frontal gyrus (6,44)	50	-4	44	5.61
	155	L/R	Medial frontal gyrus (6)	11	-4	59	5.08
FB 3	1628	R	Precentral gyrus (6), anterior insula, inferior frontal gyrus (6,44), medial frontal gyrus (6)	38	-13	38	8.37
	234	L	Anterior insula, inferior frontal gyrus (44)	-40	2	5	4.63
	147	R	Postcentral gyrus (2), inferior parietal lobe (40)	65	-28	41	4.27
	133	L	Middle frontal gyrus (6), precentral gyrus (6)	-28	-7	38	4.89
FB 4	3925	L/R	Middle frontal gyrus/precentral gyrus (6), anterior insula, medial frontal gyrus (6), inferior frontal gyrus (6,9), thalamus, putamen	38	-7	44	7.50
	283	L	Middle occipital gyrus (19), middle temporal gyrus (39)	32	-67	11	5.75
	240	L	Middle occipital gyrus (19), superior temporal gyrus (39)	-31	-67	8	4.85
XSFR	None					

### ROI ANALYSIS

**FIGURE 4:**
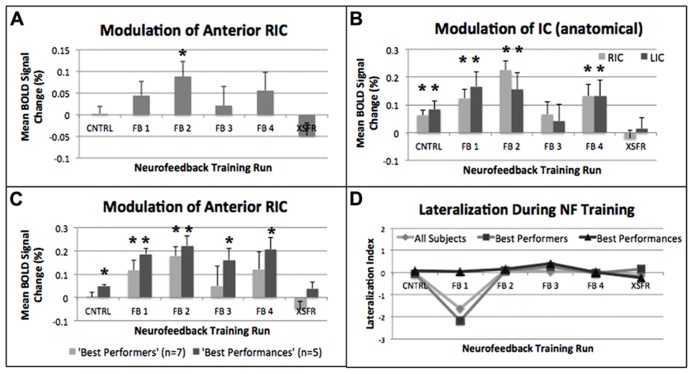
****Mean percent BOLD signal changes during each of the neurofeedback training runs showing significant (*p ≤ 0.05) increases within **(A)** the functionally localized RIC-ROI, **(B)** the structurally defined anatomical right and left anterior insular gyrus (see text), and **(C)** the functionally localized RIC-ROI of the “best performers” (*n* = 7), and the “best performances” (*n* = 5). **(D) **Calculation of a Lateralization Index (see text) using the structurally defined anterior insular gyrus showed no lateralization (+ = right; - = left) when evaluating all subjects, the “best performers,” and the “best performances.” Error bars shown are standard errors of the mean. RIC, right insular cortex; ROI, region of interest.

Mean percent BOLD signal change during rtfMRI-guided neurofeedback was significantly increased in the functionally localized RIC-ROI at the group (*n* = 14) level during the FB2 training run (*p* = 0.014, **Figure 4A**). Additionally, there was no significant effect for the treatment condition of neurofeedback training (repeated measures ANOVA, *F*(2,24) = 2.39, *p* = 0.11). Using the atlas-defined structural anterior insular cortex ROIs, activation during neurofeedback training was significantly increased bilaterally during the CNTRL (left: *p* = 0.033; right: *p* = 0.01), FB1 (left: *p* = 0.034; right: *p* = 0.004), FB2 (left: *p* = 0.004; right: *p* = 0.012), and FB4 (left: *p* = 0.009; right: *p* = 0.02), training runs (**Figure 4B**). Sub-group analysis of the “best performers” showed significant increases in RIC-ROI during the FB1 (*p* = 0.02) and FB2 (*p* = 0.002) training runs. Sub-group analysis of the “best performances” revealed significant increases during the CNTRL (*p* = 0.001), FB1 (*p* = 0.001), FB2 (*p* = 0.004), FB3 (*p* = 0.015), and FB4 (*p* = 0.009) training runs (**Figure 4C**). No significant increases in RIC-ROI were observed in the final XSFR run for either the “best performers” or the “best performances.” Although voxel-wise imaging analysis revealed greater significant cluster sizes in the region of the right compared to left insular cortex, group-level and sub-group ROI analyses showed no lateralization in the insular cortex activations during any of neurofeedback training runs (**Figure 4D**).

**FIGURE 5:**
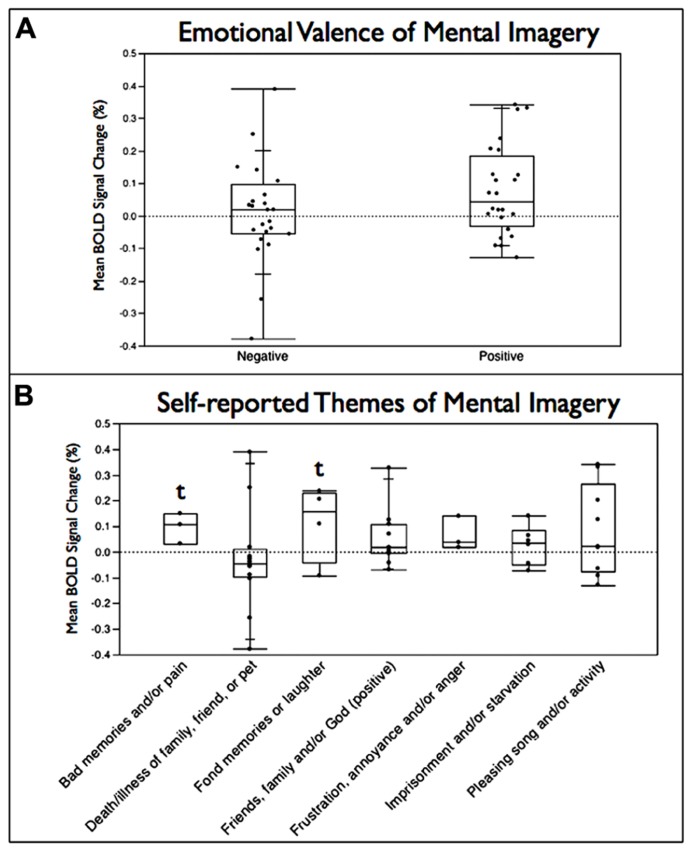
**Box plots of mean percent BOLD signal change in the functionally localized anterior RIC-ROI grouped (A) by emotional valence of the mental imagery employed by subjects during the neurofeedback training runs and **(B)** by major themes for the mental imagery seen.** Box is split at the median value and extends one quartile above and below the median; lines extend to maximum and minimum values in the distribution (*t* = statistical trend, *p* < 0.06).

The emotional valence of the mental imagery used by subjects was unable to be assessed for 33 of the total of 81 usable neurofeedback runs across subjects due to insufficient detail in the self-reported summaries provided by subjects. Comparing the runs where positive (*n* = 24) and negative (*n* = 24) valence could be ascribed to the type mental strategy employed, no significant difference (*p *> 0.05) was observed between imagery with positive and negative valence in leading to a greater BOLD increase within RIC-ROI (**Figure 5A**). Self-reported mental imagery topics were then grouped into seven major themes with a trend toward significant increases observed for mental strategies involving bad memories and/or pain (*p* = 0.052) and positive thoughts of friends, family, and/or God (*p* = 0.058; **Figure 5B**).

### CONNECTIVITY ANALYSIS

**FIGURE 6:**
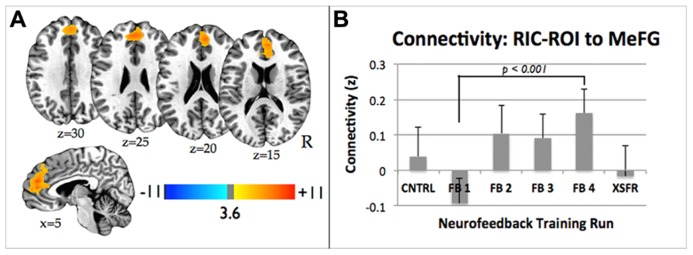
**(A)** Statistical parametric maps showing significant increase in functional connectivity between the functionally localized anterior RIC-ROI and the rest of the brain from the FB1 to FB4 training run. Images are shown at *p* ≤ 0.01, FWE corrected, on axial and sagittal slices of a standard brain in Talairach space. **(B)** Connectivity changes between RIC-ROI and the medial frontal gyrus during each of neurofeedback training runs showing significant increase in connectivity between FB1 and FB4 (*p* < 0.0001). Error bars shown are standard errors of the mean. RIC, right insular cortex; ROI, region of interest.

Neurofeedback training across FB runs resulted in an increase in functional connectivity between the RIC-ROI and medial frontal gyrus (**Figure 6A**; cluster size = 617, maximum at: -13, 35, 40). This cluster of significantly increased connectivity included a small portion of the anterior cingulate cortex. The medial frontal gyrus cluster showed a significant effect for the treatment condition (repeated measures ANOVA, *F*(3,27) = 4.83, *p* = 0.010), with increasing connectivity seen across successive training runs that disappeared for the XSFR run (**Figure 6B**). A significant increase in connectivity between RIC-ROI and the medial frontal gyrus was observed across the neurofeedback training runs with visual feedback (FB4 vs. FB1; *p* < 0.0001), but not between the CNTRL run and XSFR run (*p* = 0.56). There were no significant differences in connectivity detected between the CNTRL and XSFR runs.****

## DISCUSSION

In this study we aimed to investigate whether healthy controls could learn to modulate brain activity within a functionally localized region of their anterior RIC after a set of brief rtfMRI-based neurofeedback training sessions. At the group-level, increasing brain activity, as measured using the BOLD signal, within the RIC during feedback training was achieved. This is consistent with a number of prior studies suggesting healthy subjects can learn to use neurofeedback to increase BOLD signal in this area during a short training period (Caria et al., 2007, 2010; Johnston et al., 2010; Ruiz et al., 2011; Veit et al., 2012). Participants in our study, however, were only able to increase activity within the functionally localized target RIC-ROI during the second FB training run (FB2). Additionally, participants did not show a training effect over the four training FB runs nor did they show they achieve a learning effect as measured by the XSFR run performed following the neurofeedback training.

The limited ability of subjects to increase activation within the functionally localized RIC could stem from the cross-model nature by which the ROI was localized. Like many other brain regions, the insular cortex consists of a series of its own somatic representations (Baumgärtner et al., 2010; Stephani et al., 2011; Mazzola et al., 2012). Thus, those regions of the insular cortex localized through a motor suppression task may not be able to be modulated through the recall of emotional thoughts. Nevertheless, we observed a significant increase in activity within the target ROI during one of the FB runs. This suggests that insular regions associated with abnormal urges or behavior suppression may be able to be modulated with distinct mental imagery. By expanding on this preliminary work, the therapeutic potential of rtfMRI-based neurofeedback training on conditions with dysfunctional suppression such as Tourette syndrome or obsessive–compulsive disorder could be explored.

Why participants failed to increase activity within the target ROI in training runs following the second FB training run is not known. One possibility is that participants switched to less effective cognitive strategies around the time of this third scanning run. In post-run questioning, only four of the 14 subjects reported using the same thoughts as the prior run so a majority did switch the content of their mental imagery. Another possibility is that there may be some blunting of the brain’s emotional circuitry with sustained focus on emotionally relevant thoughts. Arguing against this explanation is our use of a task paradigm that is similar to prior rtfMRI-based neurofeedback studies that did not observe a drop in performance following a third overall training run (Caria et al., 2007, 2010). Other positive neurofeedback studies investigating the ability of subjects to modulate insular activity with emotional imagery, however, did not exceed three neurofeedback runs in a single training session (Johnston et al., 2010; Ruiz et al., 2011; Veit et al., 2012). Further study is needed to determine the ideal number of neurofeedback training runs employing emotional imagery that will optimize operant learning. This knowledge would be a particular asset to fMRI-based neurofeedback studies where scanner time can be expensive and limited.

Investigating broader BOLD changes within the RIC using group-level voxel-wise analysis and larger structurally defined ROIs, our study participants demonstrated a broader ability to increase activity during neurofeedback training. There was, however, no rightward lateralization to the insular cortex activations. The lack of lateralization to the anterior RIC may be related to the lack of specific instructions into the valence of the emotional imagery to employ during neurofeedback. Given the evidence supporting an asymmetry in emotional processing within the insular cortex (Craig, 2005), more directed content guidance in terms of the type of emotional imagery to apply during neurofeedback may improve lateralization. In those subjects who provided details of the mental strategies used during neurofeedback runs, however, no significant difference between mental imagery with positive valence and mental imagery with negative valence was found. Additionally, no clearly superior mental strategy emerged after grouping the neurofeedback runs by the overall themes of the mental strategies employed. Although further study may help elucidate the types of mental strategies that are most effective in modulating RIC activity, the therapeutic potential of neurofeedback training involving brain regions associated with emotion processing and regulation may not require unilateral modulation of cortex or modulation of a single limbic region. Rather, it may be more important to induce clinical effects through learned neurophysiological modulation of brain areas that are part of a broader limbic network (Posse et al., 2003; Johnston et al., 2010; Ruiz et al., 2011; Zotev et al., 2011; Linden et al., 2012).

Although participants demonstrated a limited ability in learning how to self-modulate neural activity within their right anterior insular cortex, we did find that the neurofeedback training led to changes in intrinsic brain dynamics. A large cluster of significantly increased functional connectivity between the RIC-ROI and medial frontal gyrus, and to a lesser extent the anterior cingulate cortex, was seen comparing the last training run (FB4) with the first training run (FB1). The medial frontal gyrus is associated with high-level executive functions including monitoring of ongoing actions and performance outcomes, as well as adjusting behavior and learning (Ridderinkhof et al., 2004). Similarly, the anterior cingulate cortex has also been posited to play a role in error monitoring and in making subsequent adjustments in behavior (Kerns et al., 2004). The medial frontal gyrus is also a region considered a key component of the default mode network, which has been hypothesized to be involved self-referential thoughts and autobiographical memory retrieval (Fox and Raichle, 2007; Mason et al., 2007; Buckner et al., 2008). The medial frontal gyrus also plays a key role in the “mentalizing network,” which partially overlaps with the default mode network and is believed to play a role in the ability to understand and manipulate the mental states of the self and others (Frith and Frith, 2006; Mars et al., 2012). One recent study in which participants were asked to make either reflective “‘mentalizing’ or ‘physical’ judgments” about themselves or others found the anterior insula was part of a shared network when we mentalize about our selves or others (Lombardo et al., 2010). Thus, neurofeedback may enable subjects to develop greater volitional control over internal thought processes and in doing so could potentially induce changes in larger brain networks.

Alterations in functional connectivity induced by rtfMRI-based neurofeedback are increasingly being reported. In one study that involved trying to train subjects to modulate activity within their supplementary motor area, decreased connectivity between the supplementary motor area and subcortical regions including the striatum and thalamus was seen (Hampson et al., 2011). Increases in connectivity within frontal and cingulate cortices during neurofeedback of attention-related neuronal activity (Lee et al., 2012) and changes in the speeds of default mode network recovery following neurofeedback training involving the auditory cortex (Van De Ville et al., 2012) have also recently been reported. Furthermore, in a small group of schizophrenia patients, rtfMRI-based neurofeedback training of the insular cortex led to increased connectivity between the insula, medial prefrontal cortex, and amygdala when the best self-regulation training session was compared to the session with the poorest performance (Ruiz et al., 2013). Together with our connectivity results, these preliminary findings support that rtfMRI-based neurofeedback training can lead to changes in brain network connectivity and raises the intriguing possibility that this technique could be used to treat neuropsychiatric disorders known to be associated with network dysfunction (Broyd et al., 2009; Fox and Greicius, 2010; Bullmore and Sporns, 2012).

Following the neurofeedback training paradigm outlined in this study, participants did not demonstrate an ability to increase activity within the insular cortex when the visual neurofeedback signal was withheld. In fact, even by evaluating the best performers and the best performances separately, no significant increases were seen in the final XSFR run, which was designed to detect whether subjects learned how to modulate brain activity in the absence of active feedback. This is in contrast to some prior reports showing healthy subjects were able to retain an improved ability to modulate their brain activity immediately following neurofeedback training (deCharms et al., 2004; Weiskopf et al., 2004; Caria et al., 2007). The majority of rtfMRI-based neurofeedback studies reported to date, however, have lacked an assessment of whether immediately following training participants retain an improved ability to control their own brain activity. It further remains to be demonstrated whether subjects participating in rtfMRI-based neurofeedback experiments learn strategies to self-regulate brain activity that can be repeated outside the scanner environment and ultimately lead to long-lasting cognitive changes (Karbach and Schubert, 2013). Given the inherent limitations of neurofeedback training using fMRI scanners as opposed to more portable and inexpensive options such as EEG-based neurofeedback, this will need to be addressed in future rtfMRI-based studies to help drive this potentially therapeutic tool forward.

In addition to assessing whether study participants learned how to self-modulate brain activity in the insular cortex immediately following neurofeedback training, we tested whether participants were able to increase activity within the insular cortex before any training began. No significant increase in insular cortex activity was detected in our voxel-wise group analysis, but we did detect a significant increase bilaterally in our anatomical insular cortex ROI analysis. It is possible that some individuals may be able to activate their insular cortices through focused emotional imagery even without neurofeedback training. Indeed, the existence of this type of potentially intrinsic human ability has been recently exploited as an approach to testing for cognitive awareness in individuals in a vegetative state (Monti et al., 2010; Yu et al., 2013). While a number of rtfMRI-based neurofeedback investigations have included control arms in which subjects receive sham feedback (deCharms et al., 2005; Caria et al., 2007, 2010; Rota et al., 2008; Yoo et al., 2008), the presence of an inherent capacity of participants to modulate activity within particular brain regions has not been well studied. It is reasonable to propose that the use of sham feedback might actually interfere with an individual’s ability to focus their thoughts and could result in an overestimation of the effects of rtfMRI-based neurofeedback training. As such, future neurofeedback studies may benefit from the inclusion of control runs before training.

One limitation to our study, and a shared limitation with most other rtfMRI-based studies, is the limited amount of time during which participants are actually devoting to neurofeedback training. After setup and localization and anatomic scanning, subjects engaged in a total of four training runs, with each run consisting of a total of 2½ min devoted to active regulation training blocks. More effective neurofeedback training may take longer and/or repeated training sessions. Training to modulate brain rhythms with EEG biofeedback may take weeks to see significant effects. In a recent rtfMRI study, schizophrenia patients were trained to modulate brain activity in their bilateral anterior insula cortices using a training paradigm that consisted of three training runs per day for four days spread out over 2 weeks (Ruiz et al., 2011). Despite this more rigorous training paradigm, patients were unable to demonstrate an ability to increase insular activity in the absence of feedback information at the end of the fourth training day. Unfortunately this study did not have a control group so it is unknown if similar findings would be seen in healthy subjects. Our study was also limited by the lack of a control group. Although more important when investigating the clinical effects of neurofeedback as therapeutic intervention, the incorporation of one or more control groups that undergo a similar training regimen while receiving no and sham feedback could help better determine the specific effect providing neurofeedback has on individuals learning to modulate brain activity.

## Conflict of Interest Statement

The authors declare that the research was conducted in the absence of any commercial or financial relationships that could be construed as a potential conflict of interest.

## Abbreviations

AFNI: Analysis of Functional NeuroImages
BOLD: blood-oxygen level dependent
CNTRL: “control” scanning run
FB: “feedback” scanning run
FOV: field of view
RIC: right insular cortex
ROI: region of interest
rtfMRI: real-time functional MRI
XSFR: “transfer” scanning run

## References

[B1] Baumgärtner U. Iannetti G. Zambreanu L. Stoeter P. Treede R. Tracey I. (2010). Multiple somatotopic representations of heat and mechanical pain in the operculo-insular cortex: a high-resolution fMRI study. *J. Neurophysiol.* 104 2863–2872 10.1152/jn.00253.2010 20739597 PMC2997041

[B2] Berman B. Horovitz S. Morel B. Hallett M. (2012a). Neural correlates of blink suppression and the buildup of a natural bodily urge. *Neuroimage* 59 1441–1450 10.1016/j.neuroimage.2011.08.050 21906689 PMC3230735

[B3] Berman B. Horovitz S. Venkataraman G. Hallett M. (2012b). Self-modulation of primary motor cortex activity with motor and motor imagery tasks using real-time fMRI-based neurofeedback. *Neuroimage* 59 917–925 10.1016/j.neuroimage.2011.07.035 21803163 PMC3222744

[B4] Bohlhalter S. Goldfine A. Matteson S. Garraux G. Hanakawa T. Kansaku K. (2006). Neural correlates of tic generation in Tourette syndrome: an event-related functional MRI study. *Brain* 129 2029–2037 10.1093/brain/awl050 16520330

[B5] Broyd S. Demanuele C. Debener S. Helps S. James C. Sonuga-Barke E. (2009). Default-mode brain dysfunction in mental disorders: a systematic review. *Neurosci. Biobehav. Rev.* 33 279–296 10.1016/j.neubiorev.2008.09.002 18824195

[B6] Buckner R. L. Andrews-Hanna J. R. Schacter D. L. (2008). The brain’s default network – anatomy, function, and relevance to disease. *Ann. N. Y. Acad. Sci.* 1124 1–38 10.1196/annals.1440.011 18400922

[B7] Bullmore E. Sporns O. (2012). The economy of brain network organization. *Nat. Rev. Neurosci.* 13 336–349 10.1038/nrn3214 22498897

[B8] Caria A. Sitaram R. Veit R. Begliomini C. Birbaumer N. (2010). Volitional control of insula activity modulates the response to aversive stimuli. A real-time functional magnetic resonance study. *Biol. Psychiatry* 68 425–432 10.1016/j.biopsych.2010.04.020 20570245

[B9] Caria A. Veit R. Sitaram R. Lotze M. Weiskopf N. Grodd W. (2007). Regulation of anterior insular cortex activity using real-time fMRI. *Neuroimage* 35 1238–1246 10.1016/j.neuroimage.2007.01.018 17336094

[B10] Cox R. W. (1996). AFNI: software for analysis and visualization of functional magnetic resonance neuroimages. *Comput. Biomed. Res.* 29 162–173 10.1006/cbmr.1996.0014 8812068

[B11] Craig A. D. (2002). How do you feel? Interoception: the sense of the physiological condition of the body. *Nat. Rev. Neurosci.* 3 655–666 10.1038/nrn894 12154366

[B12] Craig A. D. (2005). Forebrain emotional asymmetry: a neuroanatomical basis? *Trends Cogn. Sci.* 9 566–571 10.1016/j.tics.2005.10.005 16275155

[B13] Critchley H. D. Wiens S. Rotshtein P. Ohman A. Dolan R. J. (2004). Neural systems supporting interoceptive awareness. *Nat. Neurosci.* 7 189–195 10.1038/nn1176 14730305

[B14] deCharms R. C. (2007). Reading and controlling human brain activation using real-time functional magnetic resonance imaging. *Trends Cogn. Sci.* 11 473–481 10.1016/j.tics.2007.08.014 17988931

[B15] deCharms R. C. Christoff K. Glover G. H. Pauly J. M. Whitfield S. Gabrieli J. D. (2004). Learned regulation of spatially localized brain activation using real-time fMRI. *Neuroimage* 21 436–443 10.1016/j.neuroimage.2003.08.041 14741680

[B16] deCharms R. C. Maeda F. Glover G. H. Ludlow D. Pauly J. M. Soneji D. (2005). Control over brain activation and pain learned by using real-time functional MRI. *Proc. Natl. Acad. Sci. U.S.A.* 102 18626–18631 10.1073/pnas.0505210102 16352728 PMC1311906

[B17] Fahim C. Yoon U. Sandor P. Frey K. Evans A. C. (2009). Thinning of the motor-cingulate-insular cortices in siblings concordant for Tourette syndrome. *Brain Topogr.* 22 176–184 10.1007/s10548-009-0105-6 19779823

[B18] Fox M. Greicius M. (2010). Clinical applications of resting state functional connectivity. *Front. Syst. Neurosci.* 4:19 10.3389/fnsys.2010.00019 PMC2893721 20592951

[B19] Fox M. D. Raichle M. E. (2007). Spontaneous fluctuations in brain activity observed with functional magnetic resonance imaging. *Nat. Rev. Neurosci.* 8 700–711 10.1038/nrn2201 17704812

[B20] Frith C. Frith U. (2006). The neural basis of mentalizing. *Neuron* 50 531–534 10.1016/j.neuron.2006.05.001 16701204

[B21] Haller S. Birbaumer N. Veit R. (2010). Real-time fMRI feedback training may improve chronic tinnitus. *Eur. Radiol.* 20 696–703 10.1007/s00330-009-1595-z 19760238

[B22] Hampson M. Scheinost D. Qiu M. Bhawnani J. Lacadie C. Leckman J. (2011). Biofeedback of real-time functional magnetic resonance imaging data from the supplementary motor area reduces functional connectivity to subcortical regions. *Brain Connect.* 1 91–98 10.1089/brain.2011.0002 22432958 PMC3621512

[B23] Heinrich H. Gevensleben H. Strehl U. (2007). Annotation: neurofeedback - train your brain to train behaviour. *J. Child Psychol. Psychiatry* 48 3–16 10.1111/j.1469-7610.2006.01665.x 17244266

[B24] Herringa R. Phillips M. Almeida J. Insana S. Germain A. (2012). Post-traumatic stress symptoms correlate with smaller subgenual cingulate, caudate, and insula volumes in unmedicated combat veterans. *Psychiatry Res.* 203 139–145 10.1016/j.pscychresns.2012.02.005 23021615 PMC3466380

[B25] Jeffries K. J. Schooler C. Schoenbach C. Herscovitch P. Chase T. N. Braun A. R. (2002). The functional neuroanatomy of Tourette’s syndrome: an FDG PET study III: functional coupling of regional cerebral metabolic rates. *Neuropsychopharmacology* 27 92–104 10.1016/S0893-133X(01)00428-6 12062910

[B26] Johnston S. J. Boehm S. G. Healy D. Goebel R Linden D. E. J. (2010). Neurofeedback: a promising tool for the self-regulation of emotion networks. *Neuroimage* 49 1066–1072 10.1016/j.neuroimage.2009.07.056 19646532

[B27] Karbach J. Schubert T. (2013). Training-induced cognitive and neural plasticity. *Front. Hum. Neurosci.* 7:48 10.3389/fnhum.2013.00048 PMC3579194 23437015

[B28] Kerns J. Cohen J. MacDonald A. Cho R. Stenger V. Carter C. (2004). Anterior cingulate conflict monitoring and adjustments in control. *Science* 303 1023–1026 10.1126/science.1089910 14963333

[B29] Kim K. Ku J. Lee J. Lee H. Jung Y. (2012). Functional and effective connectivity of anterior insula in anorexia nervosa and bulimia nervosa. *Neurosci. Lett.* 521 152–157 10.1016/j.neulet.2012.05.075 22684096

[B30] Kwak C. Dat Vuong K. Jankovic J. (2003). Premonitory sensory phenomenon in Tourette’s syndrome. *Mov. Disord.* 18 1530–1533 10.1002/mds.10618 14673893

[B31] Lawson E. Holsen L. Santin M. Meenaghan E. Eddy K. Becker A. (2012). Oxytocin secretion is associated with severity of disordered eating psychopathology and insular cortex hypoactivation in anorexia nervosa. *J. Clin. Endocrinol. Metab.* 97 E1898-E1908 10.1210/jc.2012-1702 22872688 PMC3674290

[B32] Lee J. Kim J. Yoo S. (2012). Real-time fMRI-based neurofeedback reinforces causality of attention networks. *Neurosci. Res.* 72 347–354 10.1016/j.neures.2012.01.002 22285603

[B33] Lerner A. Bagic A. Boudreau E. A. Hanakawa T. Pagan F. Mari Z. (2007). Neuroimaging of neuronal circuits involved in tic generation in patients with Tourette syndrome. *Neurology* 68 1979–1987 10.1212/01.wnl.0000264417.18604.12 17548547

[B34] Linden D. Habes I. Johnston S. Linden S. Tatineni R. Subramanian L. (2012). Real-time self-regulation of emotion networks in patients with depression. *PLoS ONE* 7:e38115 10.1371/journal.pone.0038115 PMC3366978 22675513

[B35] Lombardo M. Chakrabarti B. Bullmore E. Wheelwright S. Sadek S. Suckling J. (2010). Shared neural circuits for mentalizing about the self and others. *J. Cogn. Neurosci.* 22 1623–1635 10.1162/jocn.2009.21287 19580380

[B36] Mars R. Neubert F. Noonan M. Sallet J. Toni I. Rushworth M. (2012). On the relationship between the “default mode network” and the “social brain”. *Front. Hum. Neurosci.* 6:189 10.3389/fnhum.2012.00189 PMC3380415 22737119

[B37] Mason M. Norton M. Van Horn J. Wegner D. Grafton S. Macrae C. (2007). Wandering minds: the default network and stimulus-independent thought. *Science* 315 393–395 10.1126/science.1131295 17234951 PMC1821121

[B38] Mazzola L. Faillenot I. Barral F. Mauguière F. Peyron R. (2012). Spatial segregation of somato-sensory and pain activations in the human operculo-insular cortex. *Neuroimage* 60 409–418 10.1016/j.neuroimage.2011.12.072 22245639

[B39] Messerotti Benvenuti S. Buodo G. Leone V. Palomba D. (2011). Neurofeedback training for tourette syndrome: an uncontrolled single case study. *Appl. Psychophysiol. Biofeedback* 36 281–288 10.1007/s10484-011-9169-7 21915704

[B40] Monti M. Vanhaudenhuyse A. Coleman M. Boly M. Pickard J. Tshibanda L. (2010). Willful modulation of brain activity in disorders of consciousness. *N. Engl. J. Med.* 362 579–589 10.1056/NEJMoa0905370 20130250

[B41] Nagai M. Kishi K. Kato S. (2007). Insular cortex and neuropsychiatric disorders: a review of recent literature. *Eur. Psychiatry* 22 387–394 10.1016/j.eurpsy.2007.02.006 17416488

[B42] Nishida S. Narumoto J. Sakai Y. Matsuoka T. Nakamae T. Yamada K. (2011). Anterior insular volume is larger in patients with obsessive–compulsive disorder. *Prog. Neuropsychopharmacol. Biol. Psychiatry* 35 997–1001 10.1016/j.pnpbp.2011.01.022 21303681

[B43] O’Connor K. Gareau D. Borgeat F. (1995). Muscle control in chronic tic disorders. *Biofeedback Self Regul.* 20 111–122 10.1007/BF01720968 7662748

[B44] Oldfield R. C. (1971). Assessment and analysis of handedness – Edinburgh inventory. *Neuropsychologia* 9 97–113 10.1016/0028-3932(71)90067-4 5146491

[B45] Peterson B. S. Leckman J. F. (1998). The temporal dynamics of ties in Gilles de la Tourette syndrome. *Biol. Psychiatry* 44 1337–1348 10.1016/S0006-3223(98)00176-0 9861477

[B46] Phan K. L. Wager T. Taylor S. F. Liberzon I. (2002). Functional neuroanatomy of emotion: a meta-analysis of emotion activation studies in PET and fMRI. *Neuroimage* 16 331–348 10.1006/nimg.2002.1087 12030820

[B47] Piacentini J. Chang S. (2001). Behavioral treatments for Tourette syndrome and tic disorders: state of the art. *Adv. Neurol.* 85 319–331 11530440

[B48] Posse S. Binkofski F. Schneider F. Gembris D. Frings W. Habel U. (2001). A new approach to measure single-event related brain activity using real-time fMRI: feasibility of sensory, motor, and higher cognitive tasks. *Hum. Brain Mapp.* 12 25–41 10.1002/1097-0193(200101)12:1<25::AID-HBM30>3.0.CO;2-H 11198103 PMC6871962

[B49] Posse S. Fitzgerald D. Gao K. Habel U. Rosenberg D. Moore G. J. (2003). Real-time fMRI of temporolimbic regions detects amygdala activation during single-trial self-induced sadness. *Neuroimage* 18 760–768 10.1016/S1053-8119(03)00004-1 12667853

[B50] Ridderinkhof K. Ullsperger M. Crone E. Nieuwenhuis S. (2004). The role of the medial frontal cortex in cognitive control. *Science* 306 443–447 10.1126/science.1100301 15486290

[B51] Rota G. Sitaram R. Veit R. Erb M. Weiskopf N. Dogil G. (2008). Self-regulation of regional cortical activity using real-time fMRI: the right inferior frontal gyrus and linguistic processing. *Hum. Brain Mapp.* 30 1605–1614 10.1002/hbm.20621 18661503 PMC6870622

[B52] Ruiz S. Lee S. Soekadar S. R. Caria A. Veit R. Kircher T. (2011). Self-control of insula cortex by real-time fMRI modulates face emotion recognition and brain network connectivity in schizophrenia. *Psychophysiology* 48 S90–S90 10.1002/hbm.21427 PMC6869886 22021045

[B53] Ruiz S. Lee S. Soekadar S. Caria A. Veit R. Kircher T. (2013). Acquired self-control of insula cortex modulates emotion recognition and brain network connectivity in schizophrenia. *Hum. Brain Mapp.* 34 200–212 10.1002/hbm.21427 22021045 PMC6869886

[B54] Shapiro A. K. Shapiro E. S. Young J. G. Feinberg T. E. (1988). *Gilles de la Tourette Syndrome*. New York: Raven Press

[B55] Stephani C. Fernandez-Baca Vaca G. Maciunas R. Koubeissi M Lüders H. (2011). Functional neuroanatomy of the insular lobe. *Brain Struct. Funct.* 216 137–149 10.1007/s00429-010-0296-3 21153903 PMC3097350

[B56] Stern E. Silbersweig D. A. Chee K. Y. Holmes A. Robertson M. M. Trimble M. (2000). A functional neuroanatomy of tics in Tourette syndrome. *Arch. Gen. Psychiatry* 57 741–748 10.1001/archpsyc.57.8.741 10920461

[B57] Stern E. Welsh R. Fitzgerald K. Gehring W. Lister J. Himle J. (2011). Hyperactive error responses and altered connectivity in ventromedial and frontoinsular cortices in obsessive–compulsive disorder. *Biol. Psychiatry* 69 583–591 10.1016/j.biopsych.2010.09.048 21144497 PMC3059508

[B58] Subramanian L. Hindle J. V. Johnston S. Roberts M. V. Husain M. Goebel R. (2011). Real-time functional magnetic resonance imaging neurofeedback for treatment of parkinson’s disease. *J. Neurosci.* 31 16309–16317 10.1523/JNEUROSCI.3498-11.2011 22072682 PMC6633236

[B59] Talairach J. Tournoux P. (1988). *Co-Planar Stereotaxic Atlas of the Human Brain: 3-Dimensional Proportional System: An Approach to Cerebral Imaging*. New York: Thieme

[B60] Tansey M. A. (1986). A simple and a complex tic (Gilles de la Tourette’s syndrome): their response to EEG sensorimotor rhythm biofeedback training. *Int. J. Psychophysiol.* 4 91–97 10.1016/0167-8760(86)90002-4 3460976

[B61] Van De Ville D. Jhooti P. Haas T. Kopel R. Lovblad K. Scheffler K. (2012). Recovery of the default mode network after demanding neurofeedback training occurs in spatio-temporally segregated subnetworks. *Neuroimage* 63 1775–1781 10.1016/j.neuroimage.2012.08.061 22960086

[B62] Veit R. Singh V. Sitaram R. Caria A. Rauss K. Birbaumer N. (2012). Using real-time fMRI to learn voluntary regulation of the anterior insula in the presence of threat-related stimuli. *Soc. Cogn. Affect. Neurosci.* 7 623–634 10.1093/scan/nsr061 21983794 PMC3427870

[B63] Weiskopf N. (2012). Real-time fMRI and its application to neurofeedback. *Neuroimage* 62 682–692 10.1016/j.neuroimage.2011.10.009 22019880

[B64] Weiskopf N. Mathiak K. Bock S. W. Scharnowski F. Veit R. Grodd W. (2004). Principles of a brain-computer interface (BCI) based on real-time functional magnetic resonance imaging (fMRI). *IEEE Trans. Biomed. Eng.* 51 966–970 10.1109/TBME.2004.827063 15188865

[B65] Weiskopf N. Sitaram R. Josephs O. Veit R. Scharnowski F. Goebel R. (2007). Real-time functional magnetic resonance imaging: methods and applications. *Magn. Reson. Imaging* 25 989–1003 10.1016/j.mri.2007.02.007 17451904

[B66] Weiskopf N. Veit R. Erb M. Mathiak K. Grodd W. Goebel R. (2003). Physiological self-regulation of regional brain activity using real-time functional magnetic resonance imaging (fMRI): methodology and exemplary data. *Neuroimage* 19 577–586 10.1016/S1053-8119(03)00145-9 12880789

[B67] Yoo S. S. Fairneny T. Chen N. K. Choo S. E. Panych L. P. Park H. (2004). Brain-computer interface using fMRI: spatial navigation by thoughts. *Neuroreport* 15 1591–1595 10.1097/01.wnr.0000133296.39160.fe 15232289

[B68] Yoo S. S. Jolesz F. A. (2002). Functional MRI for neurofeedback: feasibility study on a hand motor task. *Neuroreport* 13 1377–1381 10.1097/00001756-200208070-00005 12167756

[B69] Yoo S. S. Lee J. H. O’Leary H. Panych L. P. Jolesz F. A. (2008). Neurofeedback fMRI-mediated learning and consolidation of regional brain activation during motor imagery. *Int. J. Imaging Syst. Technol.* 18 69–78 10.1002/ima.20139 19526048 PMC2630170

[B70] Yoo S. S. O’Leary H. M. Fairneny T. Chen N. K. Panych L. P. Park H. (2006). Increasing cortical activity in auditory areas through neurofeedback functional magnetic resonance imaging. *Neuroreport* 17 1273–1278 10.1097/01.wnr.0000227996.53540.22 16951568

[B71] Yoo S. S. O’Leary H. M. Lee J. H. Chen N. K. Panych L. P. Jolesz F. A. (2007). Reproducibility of trial-based functional MRI on motor imagery. *Int. J. Neurosci.* 117 215–227 10.1080/00207450600582546 17365109

[B72] Yu T. Lang S. Vogel D. Markl A. Müller F. Kotchoubey B. (2013). Patients with unresponsive wakefulness syndrome respond to the pain cries of other people. *Neurology* 80 345–352 10.1212/WNL.0b013e31827f0846 23255830

[B73] Zotev V. Krueger F. Phillips R. Alvarez R. P. Simmons W. K. Bellgowan P. (2011). Self-regulation of amygdala activation using real-time fMRI neurofeedback. *PLoS ONE* 6:e24522 10.1371/journal.pone.0024522 PMC3169601 21931738

